# Language profile of children with cochlear implants: comparative study about the effect of age of cochlear implantation and the duration of rehabilitation

**DOI:** 10.1007/s00405-024-08689-8

**Published:** 2024-05-16

**Authors:** Heba Mahmoud Farag, Dalia Mostafa Osman, Rasha Farouk Safwat

**Affiliations:** Phoniatrics, Phoniatric Unit, ENT Department, Faculty of Medicine, Cairo University, King Faisal Street, 300, Giza, 12511 Egypt

**Keywords:** Cochlear implant, Language profile, Age of cochlear implantation, Duration of language therapy

## Abstract

**Purpose:**

The analysis of different language domains and exploration of variables that affect the outcomes of cochlear implantation would help to document the efficacy of cochlear implantation and intervention programs. The aim of this work was to examine the language profile of children with Cochlear Implants (CI) and to assess the effect of age at the time of cochlear implantation and the impact of duration of rehabilitation on the development of linguistic abilities for cochlear implanted children.

**Methods:**

The study was conducted on 46 Arabic speaking children using unilateral CI who are receiving regular post-cochlear auditory and language rehabilitation in the phoniatrics unit, Kasr Alaini hospital. A Proficient Preschooler Language Evaluation (APPEL TOOL) was applied for the assessment of different language domains.

**Results:**

Children who received post implant rehabilitation for ≥ 2 years showed significant improvement in all subtests' scores of APPEL tool than children who received same rehabilitation for ≤ 1 year. There was no significant difference of language scores between children who have received CI before age of 3 years and those who have been implanted after age of 3 years.

**Conclusion:**

This study showed that the language profile of CI children was beneficially affected by the longer duration of therapy post implantation.

## Introduction

The approach of cochlear implant (CI) is the option that could allow children with hearing loss to develop language on par with that of normal hearing children. The reported benefits in children who received CI include enhanced levels of speech perception and of spoken language proficiency, however; the device alone does not account for the variability of linguistic abilities noted over pediatric CI users [1, 2]. Although age at implantation was widely believed to be a major factor influencing outcome after cochlear implantation, there are other factors that have been suggested to account for the variability in pediatric CI children's performance across various speech and language tasks like the strategy and duration of rehabilitation, environmental factors and intellectual abilities [3, 4].

Appropriate post-implantation auditory and language training considered major factor for improving the auditory and speech perception abilities of CI children. Proper timed rehabilitation can help patients make the greatest use of their cochlear implants [5]. Sufficient post implantation verbal therapy is the basic foundation for cochlear-implant children [6]. During rehabilitation process speech perception and production tests are important to provide valuable information on cochlear implanted children's linguistic progress [7].

Appropriate assessment reveals CI children's language development level and their equivalent language age relative to normal peers. The assessment also provides information regarding whether language development of CI children is well balanced, which promotes adoption of measures in the rehabilitation training. Speech clarity, vocabulary size, successfully imitated sentences and picture description are examples of language development indicators in CI children post rehabilitation. These indicators could be used to determine the levels of speech comprehension, verbal expression and interactive communication in CI children [8].

The open speech ability post cochlear implantation is the most important indicator of successful implantation. The course of acquiring such ability could be affected by many factors [6]. Studies have shown that many factors are likely to affect auditory and speech rehabilitation efficacy in cochlear implanted children such as the age of onset of hearing loss and age of cochlear implantation [9]. Also children who have residual hearing before CI have been suggested to better adapt to the cochlear devices after implantation, which will help them to develop post-operative open speech ability [10].

Well-organized post implantation speech training is considered as an important factor for improvement of speech recognition in CI children [6]. The auditory-verbal approach for rehabilitation of CI children requires integrating the training into daily life and focuses on the initial development of audition. It includes auditory detection, auditory attention, auditory orientation, auditory memory, auditory discrimination, auditory selection, auditory feedback and auditory conception [11].

Literature were concerned more with studying the impact of age of CI on the language achievement as a whole, however limited research focused on the specific and detailed language skills post CI rehabilitation. The age at implantation is just one of the variables that could influence language development in children with CI; one more factor that requires additional studies is the effect of the duration of rehabilitation process on the outcome of cochlear implantation. The aim of this work was to assess the different aspects of language profile of children with CI and to examine whether the age of cochlear implantation or the duration of therapy could affect the linguistic abilities of CI children.

## Material and methods

### Population of the study

The sample of this study included 46 Egyptian children who suffered from congenital bilateral severe to profound sensori-neural hearing loss since birth and underwent CI procedure, the mean age of children at the time of implantation was 3 ± 0.5 years, minimum age of implantation was 2 years and maximum age of implantation was 4.3 years. Children’s chronological ages at the time of data collection ranged from 4.5 to 5 years (mean of 4.7 ± 0.1 years) and all have the same socio-economic strata. Inclusion criteria were; children with prelingual unilateral cochlear implant and with average intellectual abilities, All children were regular in the same auditory and language intervention program applied at phoniatrics unit in Kasr Alaini hospital, Cairo University by expert phoniatricians, with the same frequency of sessions for at least 6 months duration (mean duration of therapy 23.42 ± 13.84 months) with no past history of language rehabilitation. Exclusion criteria were; children with neurological disorder, psychological disorders or with other disabilities and Children who have interrupted course of rehabilitation. Comparison regarding the development of language abilities was done between children who were implanted before the age of 3 years (22 child) and after the age of 3 years (24 child) and between children who received rehabilitation for less than 1 year (18 child) and group of children who received rehabilitation for 2 years or more (28 child). The results of language assessment of the CI children were compared to normative data of typically developed children of an equivalent age.

### Ethical considerations

This study follows the principles outlined in the Declaration of Helsinki, The protocol of the study was approved by the research ethics committee of our institute N-144-2022. Parental informed consents were obtained from all patients.

### Methodology

Detailed history taking included: the current chronological age, the age of cochlear implantation, family history of similar conditions, perinatal history, developmental history, history of childhood illness, history of hearing aids use, previous language therapy and current mean of communication. All children were subjected to a thorough clinical examination. There were no associated disorders and all children were of normal examinations.

A Proficient Preschooler Language Evaluation (APPLE TOOL) [12] was applied for cochlear implanted children in this study to assess the development of different language domains. This tool was designed to evaluate different receptive and expressive language skills for native Arabic speaking children. Not only dose APPLE tool reveal receptive and expressive scores, but it also allows accessing scores for various components of language. It is a battery of several tests that includes:

Receptive vocabulary (RV): Miscellaneous picture covering different semantic group were introduced. The items included in this subtest also vary as regards level of difficulty and degree of familiarity (Total number of items of this subtest is 55).

Linguistic concepts (LC): Evaluate the child's ability to connect words with meaning and use words to refer to concepts (Total number of items of this subtest is 48).

Sentence comprehension (SC): Evaluate the child's ability to understand orally presented sentence pairing various grammatical structures (Total number of items of this subtest is 35).

Understanding oral instructions (UOI): Evaluate the child ability to follow or orally- presented instructions, a set of sheets involving pictures of many objects are introduced.For each sheet instruction is given by assessor. The instructions included in this subtest involved, for example: Sequential instructions involving different number of items or temporal indicators (Total number of items of this subtest is 28).

Expressive vocabulary (EV): various pictures sequentially introduced and the child is asked to label each of them (Total number of items of this subtest is 84).

Expressive vocabulary_1 (EV_1): This subtest evaluates the child's ability to verbally describe functions of object (Total number of items of this subtest is 16).

Morphosyntax (MS): Evaluate the child's knowledge of grammatical rules in a sentence completion task, child is asked to complete an orally presented sentence that is related to the introduced picture stimulus (Total number of items of this subtest is 60).

Word, Phrase and Sentence Repetition (REP): Evaluate the child's ability to recall and reproduce words, phrases and sentences of varying lengths and syntactic complexity, he or she is asked to repeat sentence that are orally presented by the examiner. Verbal stimuli included in this subtest are graded in difficulty and regards both length and structure complexity (Total number of items of this subtest is 22).

In All subtests, repetitions are allowed (but only before the child produce wrong responses) except in word, phrase & sentence repetition task. Each question is graded from 0 to 1 in all subtests except for Word, Phrase and Sentence Repetition (REP) each question is graded from 0 to 4; with score 4 for repetition with no errors, 3 for repetition with one error, 2 is for repetition with two errors, 1 for repetition with three errors and 0 score for repetition with four error or more.

A pilot study was carried out on 10 children prior to the study to ensure the APPEL tool applicability to the cochlear implanted children.

### Statistical analysis

Data management and statistical analysis were performed using the Statistical Package for Social Sciences (SPSS) version 21. Numerical data were summarized using means and standard deviations. For data analysis, Welch Two-Sample t-Test was done. All tests were two-tailed. P-values < 0.05 were considered significant with a 95% confidence interval (CI).

## Results

Comparison between children with CI under study and normative data of APPLE scores for children of an equivalent age showed that there was significant difference as regard all of the linguistic abilities of APPLE tool subtests (Table 1).

**Table 1:** Comparison between the APPLE tool language scores of children with cochlear implants (CI) and normal children

APPLE subtests	Normal children	CI children	P value
	Mean ± SD	Mean ± SD	
Receptive vocabulary	50.52 ± 3.7	32.28 ± 9.41	0.001*
Linguistic concept	42.67 ± 4.84	26.60 ± 8.74	0.001*
Sentence comprehension	30.06 ± 4.57	16.04 ± 6.98	0.001*
Understanding Oral instructions	17.65 ± 5.41	6.36 ± 6.45	0.001*
Receptive language score	140.74 ± 15.49	81.28 ± 27.58	0.000*
Expressive vocabulary (EV)	64.03 ± 9.44	30.08 ± 19.90	0.001*
Expressive vocabulary_1	14.77 ± 1.63	3.64 ± 4.76	0.001*
Morphosyntax	41.22 ± 9.05	5.80 ± 6.72	0.001*
Repetition	77.44 ± 17.72	36.56 ± 10.94	0.001*
Expressive language score	175.62 ± 33.33	76.08 ± 37.75	0.000*
Total language score	316.35 ± 53.45	157.36 ± 64.41	0.000*

*SD* standard deviation

*Significant P value ≤ 0.05

The results of the comparative study about the effect of age of cochlear implantation showed that there was no significant difference between all of the linguistic abilities of children who were implanted before 3 years of age and children who were implanted after 3 years (Table 2).

**Table 2:** Comparison of the language abilities of children with cochlear implants (CI) regarding the effect of age of cochlear implantation

APPLE subtests	CI before 3 years		CI after 3 years		P value
	Mean	SD	Mean	SD	
Receptive vocabulary	29.27	9.69	32.64	7.43	0.214
Linguistic concepts	24	9.38	27.09	7.59	0.247
Sentence comprehension	13.36	4.72	17	7.92	0.078
Understanding oral instructions	5.27	5.94	5.27	5.48	0.500
Total receptive score	71.91	27.20	82	22.47	0.099
Expressive vocabulary	24.27	19.03	30.82	18.61	0.266
Expressive vocabulary- 1	2.64	4.54	3.82	4.99	0.426
Morphosyntax	4.64	6.33	5.18	6.07	0.777
Repetition	35.18	11.12	36.27	11.7	0.752
Total expressive score	66.73	37.79	76.09	35.29	0.411
Total language score	138.64	64.23	158.09	56.72	0.304

*SD* standard deviation

Children with CI who received regular language therapy for 2 years or more showed statistically significant improvement of all of their linguistic abilities than CI children who received therapy for less than 1 year (Table 3).

**Table 3:** Comparison of the language abilities of children with cochlear implants (CI) regarding the effect of the duration of therapy

APPLE subtests	Therapy ≤ 1year		Therapy ≥ 2year		P value
	Mean	SD	Mean	SD	
Receptive vocabulary	24.50	3.67	34.64	8.74	0.000*
Linguistic concepts	19.13	3.59	29.22	8.59	0.000*
Sentence comprehension	11.25	1.64	17.43	7.52	0.003*
Understanding oral instructions	1.88	1.27	7.21	6.33	0.002*
Total receptive score	56.75	6.26	88.50	25.08	0.000*
Expressive vocabulary	13.13	4.81	35.79	19.32	0.000*
Expressive vocabulary- 1	0	0	5.07	5.19	0.000*
Morphosyntax	0	0	7.71	6.24	0.000*
Repetition	30.50	7.73	38.71	11.63	0.018*
Total expressive score	43.63	9.99	87.29	37.22	0.000*
Total language score	100.38	13.15	175.79	61.25	0.000*

*SD* standard deviation

^*^Significant P value ≤ 0.05

Comparison of the duration of therapy between two age groups of children who had CI before and after 3 years showed that there was no significant difference between the duration of therapy for children who were implanted before 3 years of age and children who were implanted after 3 years (Table 4).

**Table 4:** Comparison between children who were implanted before and after 3 years of age regarding the duration of therapy after cochlear implantation (CI)

	CI before 3 years, Mean SD	CI after 3 years, Mean SD	P value	CI before 3 years, Mean SD	CI after 3 years, Mean SD
Duration of therapy	24.1 m	12.7 m	23.7 m	11.8 m	0.912

*SD* standard deviation, *m* months

## Discussion

There was a need to explore various detailed and graded language skills in CI children after regular auditory and language rehabilitation and to examine whether the duration of rehabilitation or the age of implantation could affect their language profile. In this study the APPLE tool was used because it is a formal and reliable tool that could be conventional for the assessment of Arabic speaking children after regular post implant rehabilitation, it allows obtaining scores for various components of language which can be of great help to document the efficacy of intervention programs and in turn help in designing child-centered rehabilitation program.

In this study the results of language assessment of the CI children were compared to normative data of the APPLE tool to analyze the language profile of children with CI in relation to normal hearing children with an equivalent age. The results showed that children with CI performed significantly more poorly on the subtests scores, total receptive and total expressive language scores of APPLE tool compared to normal hearing children (Table 1). These findings indicated that the language development of children with CI is still limited compared to the normal children, the CI children got limited receptive and expressive vocabulary scores and used fewer morophsyntax thus their language profile was affected by their restricted various components of language.

Though children with CI gain more experience listening with the device, they were found to have smaller receptive vocabulary results than their hearing age mates. Our results are in coincide with the study of Lund [13] as he argues that when children with CI trying to “catch up” to the vocabulary knowledge of peers with normal hearing they face a difficulty to have an average vocabulary. Most studies have found the same results of limited vocabulary scores in children with CI [14]. However some studies specified that subgroups of children with CI, have achieved a degree of vocabulary knowledge compared to normal peers or are likely to do so [15]. The limited morphosyntactic profile of children with CI in this study are in line with the study done by Abdelhamid et al. [16] they found that after 5 years of regular rehabilitation, the CI children were still have delay in many morophsyntactic abilities compared to normal children.

This study showed also that children with CI did not perform as well as normal children in the subtest of sentence comprehension which was in line with other studies that were implemented in this respect [17, 18]. Our results indicated that children with CI have difficulties in sentence comprehension which may be due to deprivation of phonemic input; furthermore their auditory sense is different from children with normal hearing. Understanding oral instructions subtest's scores for CI children in this study were significantly lower than normal results, this subtest requires the engagement of short-term verbal memory to perform a certain task and it measures how children with CI became able to handle auditory stimuli. So CI children still have insufficient such skills to perform as well as normal hearing children. Our results confirm the obtained findings of the study of Dokovic and Todorovic [19] as they found that the results of understanding verbal instructions of children with CI were statistically significantly worse than the results of normal hearing children.

The poor results of the word and sentence repetition subtest in children with CI under this study could reflect the insufficient underlying mechanisms of the repetition task which includes access and integration of word meanings, and syntactic processing. Other studies applied the sentence repetition task to examine the ability of children with CI as it involves high-level of cognitive processes, they also found that Children with CI lagged behind normal hearing mates [20].

The results of this work showed that there was no significant difference in all of the APPEL tool language components' scores between children who had CI before the age of 3 years (< 3yeras) and children who had CI after 3 years (> 3 years) (Table 2). These results could be explained by that the age of CI is not the only factor that could affect the results of language development in children with CI. Children who implanted at later age could obtain significant benefit from cochlear implantation due to other factors such as using oral communication, and longer wear of hearing aid before cochlear implantation. Other researches mentioned that language skills after CI can be affected by several variables like the age of diagnosis of hearing loss, duration of implant use, information processing skills of the child and family factors [21, 22].

Our results go in line with the study of Duchesne et al. [1], they did not report on associations between the results of specific language domains and age of children at implantation. Other researchers reported that there is still a conflict regarding the outcome of early implantation as the primary factor on language and speech development [23].

Comparison of the different language domains with regard to the effect of the duration of language therapy post cochlear implantation in this study showed that CI children with therapy duration for ≥ 2yeras performed significantly better than children with therapy duration for ≤ 1year in all of APPLE tool receptive and expressive language subtests' scores (Table 3). These results confirm how the post implant remediation process for a sufficient duration is important for CI children to enable them to learn auditory stimuli and to develop their language skills.

Our results are coinciding with the results of Ertmer and Goffman [24] who found that the expressive skills were not significantly improved for prelingual children after one year of post cochlear implantation therapy. Furthermore Van Bogaert el al [25] displayed that auditory verbal therapy post cochlear implantation considered basic requirement for children's language development, they found that children with CI who enrolled in an auditory-focused speech rehabilitation approach showed significant improvement of their speech perception performance. The advantage of appropriate duration of auditory and speech therapy after cochlear implantation in improving hearing and oral communication was emphasized by other authors [26].

However our results were in contradiction with the results of Shakrawal et al. [27] who found that children in the young implanted age group who had not speech therapy achieved better scores than the later implanted group who used speech therapy. They attributed their results to the patients' full time using of CI and the maternal education, also the frequent parents' attendance in the center post implantation helped them to participate in the everyday life rehabilitation for their children.

Comparison of the duration of therapy between two age groups of children who had CI before and after 3 years showed that both age groups received comparable duration of auditory verbal therapy with no significant difference (Table 4). This result confirms that the age of implantation has not significantly impacted language profile following cochlear implantation in our study, however the improvement of language outcomes has been reported in children who received longer duration of therapy post cochlear implantation.

More work on larger numbers of CI children is needed to confirm the effectiveness of the training on auditory and language performance and to ensure the maintenance of results by looking at the course of acquisition of all language structures over a longer period of rehabilitation. Furthermore we need to apply the APEEL tool as a detailed battery of language assessment to explore the effect of other different variables that could affect language development in CI recipient children.

## Conclusion

This study described the profile of language achievement in children with CI through the detailed and separable domains of the APPLE tool, such as vocabulary, morphology, syntax and language processing. We found no statistically significant differences of the language profile between children who have received CI before age of 3 years and those who have been implanted after age of 3years. However Children whose duration of therapy was longer (≥ 2 years) gained significantly higher scores in all of the APPLE tool subtests than children whose duration of therapy was ≤ 1year.

So the duration of auditory and language therapy could be a significant factor that influenced the development of language for CI children. The obtained findings suggest that children with CI still need longer duration of auditory and language rehabilitation post cochlear implantation to improve their different language skills and to exhibit communication skills closely approximating those of hearing age mates.

## Data Availability

The datasets used and analyzed during the current study are available from the corresponding author on reasonable request.
